# Validation of the Albanian Neck Disability Index: A Reliable and Precise Tool for Assessing Disability in Young Adults with Chronic Cervical Pain

**DOI:** 10.3390/jcm15114151

**Published:** 2026-05-28

**Authors:** Elda Zeqiri, Jasemin Todri, Orges Lena

**Affiliations:** 1 Health Science PhD Program, UCAM Universidad Católica San Antonio de Murcia, Campus de los Jerónimos, No. 135 Guadalupe, 30107 Murcia, Spain; elda_zeqiri@gmx.de; 2 ÍTEM—Innovation in Manual and Physical Therapies, Research Group, Physiotherapy Department, UCAM Universidad Católica San Antonio de Murcia, Campus de los Jerónimos, No. 135 Guadalupe, 30107 Murcia, Spain; lorges@ucam.edu

**Keywords:** Neck Disability Index, psychometric validation, chronic cervical pain, reliability, patient-reported outcome measures

## Abstract

**Background:** Chronic neck pain is a prevalent musculoskeletal condition associated with functional limitations and reduced quality of life, representing a significant public health concern. The Neck Disability Index (NDI) is one of the most widely used patient-reported outcome measures for assessing neck-related disability. Although validated in multiple languages, a comprehensive psychometric evaluation of an Albanian version in a large clinical sample has been lacking. **Methods:** A methodological cross-sectional study was conducted to translate, culturally adapt, and evaluate the psychometric properties of the Albanian version of the NDI. The translation process followed standardized forward–backward cross-cultural adaptation procedures with authorization from the MAPI Research Trust. A total of 377 individuals with chronic cervical pain completed the questionnaire. Structural validity was assessed using Principal Component Analysis (PCA). Internal consistency was evaluated using Cronbach’s alpha, and test–retest reliability was assessed using intraclass correlation coefficients (ICC). Measurement error was estimated using the Standard Error of Measurement (SEM) and Minimal Detectable Change at the 95% confidence level (MDC95) at both item and total score levels. **Results:** Sampling adequacy was excellent (KMO = 0.948; Bartlett’s test *p* < 0.001). Structural validity supported a predominantly unidimensional structure, with a dominant component explaining 64.17% of the total variance. Internal consistency was excellent (Cronbach’s α = 0.99). Test–retest reliability was outstanding, with item-level ICC values ranging from 0.980 to 0.992 and a total score ICC of 0.989. Measurement error for the total score was low (SEM = 0.94; MDC95 = 2.60), indicating that changes greater than 2.60 points reflect real change beyond measurement error. Item-level MDC95 ranged from 0.36 to 0.67. **Conclusions:** The Albanian version of the NDI demonstrates strong structural validity, excellent internal consistency, high test–retest reliability, and low measurement error in individuals with chronic cervical pain. These findings are most applicable to relatively young, convenience-based Albanian-speaking populations, reflecting the characteristics of the study sample.

## 1. Introduction

Chronic neck pain is one of the most prevalent musculoskeletal disorders globally, affecting quality of life, functional capacity, and daily activities across diverse populations. It is a leading cause of long-term disability and imposes a significant socioeconomic burden due to reduced productivity and increased healthcare utilization [1,2,3].

To comprehensively assess the impact of chronic cervical pain on functional status, patient-reported outcome measures (PROMs) have become essential tools in both clinical practice and research. Among these, the Neck Disability Index (NDI) is the most widely used instrument for quantifying self-reported neck-related disability [4,5,6].

Originally developed by Vernon and Mior in 1991, the NDI is a 10-item self-report questionnaire designed to evaluate the extent to which neck pain interferes with activities of daily living, including pain intensity, personal care, lifting, reading, work, driving, sleeping, recreation, and concentration [7]. Each item is scored on a six-point scale (0–5), with higher scores indicating greater disability. Due to its brevity, ease of administration, and responsiveness to clinical change, the NDI has been extensively adopted in musculoskeletal research and clinical settings to monitor treatment outcomes and support clinical decision-making [6].

However, the valid application of PROMs across different linguistic and cultural contexts requires rigorous translation and cross-cultural adaptation procedures. Simple translation is insufficient; semantic equivalence, cultural relevance, and conceptual consistency must be ensured to preserve the psychometric integrity of the instrument. Established guidelines recommend a multistep process, including forward translation, back-translation, expert committee review, pre-testing, and cognitive debriefing with patients [8].

The NDI has been successfully translated and validated in numerous languages and cultural settings. The simplified Chinese version demonstrated excellent internal consistency (Cronbach’s α = 0.92), good test–retest reliability (ICC = 0.85), and strong validity in patients with neck pain [9]. Similarly, the Hindi version showed high internal consistency (α = 0.90) and reliability (ICC = 0.92) in individuals with chronic neck pain [10]. The Arabic version also reported excellent reliability (ICC = 0.96) and strong internal consistency (α = 0.89), with factor analysis supporting its structural validity [11]. Additional adaptations in European Portuguese, Polish, and Japanese populations have demonstrated acceptable psychometric properties, supporting their use in both clinical and research contexts [12,13,14]. These findings highlight the robustness and cross-cultural applicability of the NDI.

Despite these advances, systematic reviews have identified variability in methodological quality across translated versions. A comprehensive review reported more than two dozen adaptations of the NDI but noted limitations related to small sample sizes, inconsistent reporting, and limited evaluation of measurement error and interpretability [15]. These findings underscore the need for rigorous and comprehensive validation studies, particularly in underrepresented linguistic populations.

In the Albanian context, a preliminary study has recently reported the translation, cross-cultural adaptation, and initial psychometric evaluation of the NDI (ANDI) [16]. This initial phase included 83 participants with neck pain and focused primarily on pre-testing, cognitive debriefing, and the assessment of internal consistency and short-term test–retest reliability over a three-day interval. The findings supported the clarity, cultural appropriateness, and preliminary reliability of the translated instrument. However, this earlier study was limited in scope and was not designed to provide a comprehensive psychometric validation.

Importantly, the present study represents a subsequent and methodologically distinct validation phase, conducted in a new and independent sample of participants, none of whom were included in the preliminary study. In contrast to the initial phase, the current investigation focuses exclusively on individuals with chronic cervical pain, providing a clinically more homogeneous and relevant population for validation. Furthermore, it extends the psychometric evaluation by examining structural validity, measurement error, and test–retest reliability over a longer interval, thereby offering a more comprehensive assessment of the instrument’s measurement properties.

Given the increasing emphasis on culturally competent healthcare and the global burden of chronic neck pain, the availability of valid and reliable PROMs in different languages is essential. Therefore, the aim of this study was to perform a comprehensive psychometric validation of the ANDI in patients with chronic cervical pain.

## 2. Methods

### 2.1. Study Design

This methodological study was conducted to translate, culturally adapt, and evaluate the psychometric properties of the ANDI in individuals with chronic cervical pain. The study design followed internationally accepted methodological standards for cross-cultural adaptation of patient-reported outcome measures (PROMs) as described by Beaton et al. [8], and adhered to the recommendations of the COSMIN (COnsensus-based Standards for the selection of health Measurement INstruments) initiative for evaluating measurement properties [17,18]. The research was conducted in two sequential phases: (1) translation and cross-cultural adaptation and (2) psychometric evaluation including reliability, validity, structural validity, and measurement error.

This study represents a subsequent and independent phase following prior translation and pre-testing work and involves a new and larger clinical sample specifically recruited for full psychometric validation [16]. No prior study has reported a full psychometric validation of the Albanian NDI in a clinical population with chronic cervical pain.

The study protocol was reviewed and approved by the Institutional Ethics Committee of UCAM Catholic University of Murcia with approval ID: CE012516, and from the Albanian Health and Social Ministry Ethics Committee (Protocol ID: 208/9). The study was prospectively registered at ClinicalTrials.gov under the identifier NCT06833359. All procedures were performed in accordance with the Declaration of Helsinki. Written informed consent was obtained from all participants prior to enrollment.

### 2.2. Participants

The present study was conducted in a new and independent sample of participants, distinct from those involved in the initial pre-testing phase [16], recruited consecutively between 25 February 2025 and 30 January 2026 through university settings and affiliated physiotherapy clinics in different cities of Albania, using a convenience sampling approach. Individuals were eligible if they were native Albanian speakers, aged 18 years or older, and had a diagnosis of chronic neck pain, defined as pain persisting for at least three months [1,2]. Chronic neck pain was identified based on clinical evaluation and patient self-report.

Exclusion criteria included previous cervical spine surgery within the past six months, diagnosed neurological disorders affecting upper limb function, systemic inflammatory or rheumatologic diseases, malignancy, or cognitive impairment preventing questionnaire completion. Consecutive sampling was applied to minimize selection bias and enhance generalizability of findings.

### 2.3. Sample Size Calculation

The sample size was determined according to COSMIN recommendations for studies evaluating measurement properties of PROMs [17,18]. For analyses of internal consistency and factor structure, it is recommended to include between 5 and 10 participants per questionnaire item, with a minimum sample size of 100 considered adequate for exploratory factor analysis [17]. Given that the NDI consists of 10 items, a target sample size of at least 100 participants was established.

For test–retest reliability assessment using the Intraclass Correlation Coefficient (ICC), calculations were based on the method described by Walter et al. [19]. Assuming an expected ICC of 0.85, a minimum acceptable ICC of 0.70, a significance level of 0.05, and statistical power of 80%, a minimum of 45 participants was required. To account for potential attrition, at least 100 participants were invited to complete the retest assessment.

### 2.4. Instrument: Neck Disability Index (NDI)

NDI is a self-administered questionnaire developed to measure disability associated with neck pain [7]. It consists of 10 items evaluating pain intensity and functional limitations across daily activities, including personal care, lifting, reading, headaches, concentration, work, driving, sleeping, and recreation. Each item is scored on a six-point Likert scale from 0 (no disability) to 5 (complete disability), yielding a total score ranging from 0 to 50. The score is commonly converted into a percentage (total score × 2), with higher scores indicating greater disability.

The NDI has demonstrated strong psychometric properties across multiple populations and languages [4,5,15]. Its widespread use and established validity make it the reference standard for assessing neck-related disability in both clinical practice and research settings.

### 2.5. Translation and Cross-Cultural Adaptation

Permission to translate and validate the NDI into Albanian was formally obtained from the MAPI Research Trust through the ePROVIDE platform ID request approval: 2423069, which manages the intellectual property rights and distribution of the instrument.

The translation and cross-cultural adaptation of the NDI into Albanian followed the standardized methodology proposed by Beaton et al. [8], which ensures semantic, idiomatic, experiential, and conceptual equivalence between the original and adapted versions.

#### 2.5.1. Forward Translation

Two independent bilingual translators whose mother tongue was Albanian performed forward translations from English into Albanian. One translator had a clinical background and was familiar with musculoskeletal terminology, ensuring conceptual equivalence. The second translator had no medical background, ensuring linguistic naturalness and lay comprehension.

#### 2.5.2. Synthesis of Translations

The two forward translations were compared and synthesized into a single preliminary version (T12) during a consensus meeting involving the translators and research team.

#### 2.5.3. Back Translation

Two independent native English speakers, blinded to the original NDI and without clinical knowledge of the instrument, translated the synthesized Albanian version back into English. This step ensured that the translated items reflected the same item content as the original questionnaire and helped identify inconsistencies or conceptual deviations.

#### 2.5.4. Expert Committee Review

An expert committee composed of physiotherapists, a rehabilitation physician, a linguist, and a methodologist reviewed all translation stages. The committee evaluated semantic equivalence (word meaning), idiomatic equivalence (colloquial expressions), experiential equivalence (relevance to Albanian culture), and conceptual equivalence (construct consistency), as recommended by international guidelines [8]. A pre-final Albanian version was then established.

#### 2.5.5. Pre-Testing and Cognitive Debriefing

The pre-final version of the ANDI was pilot tested in an independent sample of 83 individuals with neck pain, as previously reported [16]. This preliminary phase aimed to evaluate clarity, comprehensibility, and cultural relevance of the translated items through cognitive debriefing interviews. Participants completed the questionnaire at two time points, three days apart, to assess initial test–retest reliability and internal consistency.

Based on participant feedback, minor linguistic adjustments were made, and the final Albanian version (ANDI) was established. Importantly, none of the participants from this preliminary study were included in the present validation cohort, ensuring full independence between datasets.

### 2.6. Psychometric Evaluation

#### Structural Validity

Structural validity was assessed using Principal Component Analysis (PCA) with Varimax rotation to explore the underlying factor structure of the Albanian version of the Neck Disability Index. Sampling adequacy was evaluated using the Kaiser–Meyer–Olkin (KMO) measure and Bartlett’s test of sphericity [20]. Components with eigenvalues greater than 1 were retained according to Kaiser’s criterion. Factor loadings ≥ 0.40 were considered meaningful for interpretation.

### 2.7. Reliability

#### 2.7.1. Internal Consistency

Internal consistency reflects the degree to which items measure the same construct. It was evaluated using Cronbach’s alpha coefficient, with values between 0.70 and 0.95 considered acceptable [17,21]. Item-total correlations were calculated, and Cronbach’s alpha if an item was deleted was examined to detect redundant or poorly performing items.

#### 2.7.2. Test–Retest Reliability

Test–retest reliability assesses stability over time in patients whose clinical condition remains unchanged. All participants (n = 377) completed the retest assessment after a standardized interval of 7 days. Given the chronic nature of cervical pain in the study population, clinical stability was assumed over this short time period. However, no formal assessment of symptom stability (e.g., global rating of change) was performed to confirm the absence of clinical change between test administrations.

Reliability was calculated using the two-way random-effects model Intraclass Correlation Coefficient (ICC 2.1) [21]. ICC values were interpreted as follows: <0.50: poor; 0.50–0.75: moderate; 0.75–0.90: good; 0.90: excellent reliability [21].

Measurement error was quantified using the Standard Error of Measurement (SEM) and Minimal Detectable Change at the 95% confidence level (MDC95), calculated according to established formulas [17].

### 2.8. Statistical Analysis

Data analysis was performed using SPSS version 25 (IBM Corp., Armonk, NY, USA) [20]. Descriptive statistics were calculated for demographic and clinical characteristics. Continuous variables were expressed as mean ± standard deviation or median (interquartile range), depending on data distribution. Normality was assessed using the Shapiro–Wilk test. Pearson or Spearman correlation coefficients were used as appropriate. Statistical significance was set at *p* < 0.05.

## 3. Results

A total of 377 participants with chronic neck pain were included in the final analysis. The mean age of the sample was 30.08 ± 12.97 years (range: 18–72), indicating a relatively young but heterogeneous adult population. The mean body weight was 67.56 ± 13.98 kg (range: 40–125), and the mean height was 168.65 ± 8.57 cm (range: 150–191).

The majority of participants were female (69.8%, n = 263), while males accounted for 30.2% (n = 114) (Table 1).

Regarding occupational distribution, students represented the largest subgroup (30.5%, n = 115), followed by physiotherapists (11.7%, n = 44), economists (7.4%, n = 28), and managers (6.9%, n = 26). Healthcare-related professions were notably represented, while several other occupations appeared at lower frequencies (<5%), reflecting a convenience-based and functionally active sample (Table 1).

### 3.1. Psychometric Properties of the Albanian NDI

#### 3.1.1. Structural Validity

Structural validity analysis demonstrated excellent sampling adequacy (KMO = 0.947) and a significant Bartlett’s test of sphericity (χ^2^ = 2595.798, df = 45, *p* < 0.001), confirming suitability for factor analysis.

PCA identified a single dominant component (eigenvalue = 6.417), explaining 64.17% of the total variance. Only this component exceeded the Kaiser criterion, supporting a unidimensional structure.

All items demonstrated strong loadings (0.690–0.876), indicating substantial coherence across items and supporting the measurement of a common construct of neck-related disability. Table 2 displays the structural validity results.

#### 3.1.2. Internal Consistency

Internal consistency of the ANDI was very high. Corrected item–total correlations ranged from 0.962 to 0.990, indicating strong associations between individual items and the overall score.

All items exceeded accepted thresholds, confirming their contribution to a common construct. The narrow range of correlations suggests a high degree of homogeneity across items rather than variability in item performance.

Item-level descriptive statistics showed moderate mean values (0.72–1.53), reflecting distribution across the sample.

No item demonstrated a meaningfully weaker association with the total score, supporting retention of all items (Table 3).

#### 3.1.3. Test–Retest Reliability

Test–retest reliability was assessed using a two-way random-effects model with absolute agreement.

Item-level ICC values ranged from 0.980 to 0.992 (*p* < 0.001), indicating excellent temporal stability over the 7-day interval. The narrow 95% confidence intervals support the precision of these estimates (Table 3).

All participants completed both assessments under identical conditions.

The total NDI score also demonstrated excellent reliability (ICC = 0.989), confirming stability at the scale level.

#### 3.1.4. Measurement Error

Measurement error was low at both item and total score levels.

At the item level (0–5 scale), MDC_95_ ranged from 0.36 to 0.67 points. Although small differences were observed across items, all values indicate minimal measurement variability.

The lowest error was observed for Sleeping and Reading, while Driving and Lifting showed slightly higher—but still clinically small—values (Table 3).

At the total score level, SEM was 0.94 and MDC_95_ was 2.60 points, indicating that a change greater than 2.60 points reflects a real change beyond measurement error.

## 4. Discussion

The present study evaluated the psychometric properties of the ANDI in individuals with chronic cervical pain. Overall, the findings consistently demonstrate strong measurement properties, including unidimensional structure, very high internal consistency, excellent reliability, and low measurement error. These results are in line with previous cross-cultural validations.

### 4.1. Structural Validity

The findings support a primarily unidimensional structure of the ANDI. The strong KMO value and significant Bartlett’s test confirmed the adequacy of the data for factor analysis.

A single dominant component explained over 64% of the variance, supporting the interpretation of the NDI as a unified measure of neck-related disability.

Although multidimensional structures have been reported in some studies [22,23,24,25,26], the dominance of a single component in this study, combined with consistent item loadings, supports the use of a total score in clinical and research contexts.

The original English validation conceptualized the NDI as a unidimensional scale measuring neck-related disability [7]. However, subsequent cross-cultural validations have reported both one- and two-factor models. The Spanish version identified a two-factor structure separating physical function from pain-related symptoms [22]. Similarly, the Turkish validation demonstrated a two-component solution reflecting symptom intensity and daily activities [23]. The Persian version also reported a multidimensional construct while maintaining strong overall factorial coherence [24].

More recent validations, such as the Italian [25] and Polish [26] versions, have suggested that although statistical analyses may extract more than one factor, the scale behaves clinically as a unidimensional construct.

The explained variance observed in this study was slightly higher than previously reported ranges (48–62%) [23,24,25,26,27], which may reflect the relative homogeneity of the chronic pain sample.

### 4.2. Internal Consistency

The present study demonstrated extremely high internal consistency (Cronbach’s α = 0.99), which exceeds commonly accepted thresholds for excellent reliability. While this finding indicates a high degree of interrelatedness among items, such elevated coefficients may also suggest potential item redundancy, as items may be capturing highly overlapping aspects of the construct.

The ANDI demonstrated extremely high internal consistency (α = 0.99). While this confirms strong interrelatedness among items, such high values may also indicate redundancy.

Rather than reflecting independent domains, items appear to capture closely related aspects of disability, which is consistent with the conceptual structure of the NDI. Similar patterns have been reported in previous studies [4,11,25,26,27]. Reported Cronbach’s alpha values typically range between 0.80 and 0.95 [4].

Importantly, removal of any item did not improve reliability, supporting retention of the original structure.

### 4.3. Test–Retest Reliability

The ANDI demonstrated excellent temporal stability, with item-level ICC values ranging from 0.980 to 0.992 and total score ICC = 0.989. These findings indicate high reproducibility in stable conditions.

Comparable results have been reported in Spanish (ICC = 0.97) [22], Turkish (ICC = 0.92) [23], Persian (ICC = 0.97) [24], Italian (ICC = 0.94) [25], and Arabic versions (>0.90) [11].

According to COSMIN criteria, these values indicate excellent reliability [18].

The consistency between item-level and total score reliability strengthens confidence in the stability of the instrument.

#### 4.3.1. Measurement Error

Measurement error was low, supporting the precision of the instrument.

At the total score level, MDC_95_ was 2.60 points, which is lower than values reported in previous studies (3–7 points) [6,28].

This suggests that the ANDI may be sensitive to relatively small changes, particularly in similar populations.

However, differences across studies may be influenced by sample characteristics, scoring approaches, and variability.

While low MDC_95_ indicates strong measurement precision, responsiveness was not directly assessed, and future longitudinal studies are needed.

#### 4.3.2. Comparison with International Evidence

The present study should be interpreted in the context of prior preliminary work conducted on the Albanian version of the NDI. While the earlier study confirmed initial reliability and internal consistency in a small sample (n = 83), it primarily served as a pilot phase following translation and cross-cultural adaptation [16]. In contrast, the current study represents the first large-scale validation conducted in an independent cohort of patients with chronic cervical pain.

Importantly, the samples in the two studies were completely independent, and the present analysis includes additional psychometric evaluations, such as structural validity and measurement error, which were not addressed previously. This sequential approach is consistent with recommended methodological frameworks for the development and validation of patient-reported outcome measures, where initial pre-testing is followed by full psychometric validation in a larger and clinically relevant population.

The findings align with prior NDI validation studies across multiple languages [8,23,24,25,26,27].

The consistency of high reliability and validity across cultures supports the robustness of the NDI as a measurement tool.

The slightly higher reliability estimates observed in this study may reflect the chronic nature of the sample, as stable symptom patterns typically produce higher consistency [6].

## 5. Clinical Implications

The ANDI provides a reliable and precise tool for assessing neck disability in Albanian-speaking individuals.

Its strong measurement properties support its use in clinical monitoring and research, particularly in populations similar to the present sample.

Furthermore, the preservation of structural properties comparable to other language versions enhances its suitability for international multicenter research.

## 6. Limitations and Future Directions

Despite the robust psychometric findings, several limitations should be acknowledged. Structural validity was assessed using EFA without complementary hypothesis-driven testing against external comparator instruments. Therefore, broader aspects of construct validity such as convergent and discriminant validity could not be evaluated. Future studies should incorporate established patient-reported outcome measures (pain intensity scales or quality-of-life instruments) to enable hypothesis testing in accordance with COSMIN recommendations. In addition, CFA would be valuable to further examine and validate the dimensional structure identified in this study.

The generalizability of the findings may be influenced by the characteristics of the study sample. The mean age of participants was relatively young, and a substantial proportion consisted of students and physiotherapists. This composition may reflect a more health-aware and functionally active subgroup of individuals with chronic neck pain, potentially limiting the representativeness of the sample. Consequently, the results may not fully generalize to older populations, individuals with more severe disability, or patients with complex clinical profiles and comorbid conditions. Future research should aim to validate the ANDI in more diverse and clinically representative populations, including older adults and patients recruited from specialized healthcare settings.

Although test–retest reliability demonstrated excellent stability, its interpretation warrants caution. Clinical stability between test administrations was assumed based on the chronic nature of the condition and the relatively short 7-day interval; however, no formal method (such as a global rating of change or symptom stability assessment) was used to confirm that participants’ clinical status remained unchanged. Future studies should incorporate explicit measures of stability to strengthen the methodological rigor of reliability assessment.

Even though measurement error was quantified through the SEM and MDC_95_, responsiveness and MCID were not evaluated in this study. As such, the ability of the instrument to detect clinically meaningful change over time remains to be established. Longitudinal studies are needed to assess responsiveness and to define clinically interpretable change thresholds. Additionally, future research could explore advanced psychometric approaches, such as item response theory (IRT) modeling, to further refine measurement precision and evaluate item-level performance.

The characteristics of the study sample should also be considered when interpreting the findings. The mean age of participants was relatively young (30.08 years), and a substantial proportion consisted of students and healthcare-related professionals, including physiotherapists. This may limit the generalizability of the results to the broader population of individuals with chronic neck pain, particularly older adults, individuals with more severe disability, or patients recruited from specialized clinical settings. Therefore, while the psychometric properties observed in this study are robust, caution is warranted when extending these findings beyond similar populations.

## 7. Conclusions

The ANDI demonstrates strong structural validity, excellent reliability, and low measurement error. These findings are most applicable to relatively young, convenience-based populations with chronic neck pain.

Further research is needed to confirm these results in broader clinical populations.

## Figures and Tables

**Table 1:** Demographic and clinical characteristics of the participants.

Characteristics	Total Sample (n = 377)
Age (years)	30.08 ± 12.97 (18–72)
Weight (kg)	67.56 ± 13.98 (40–125)
Height (cm)	168.65 ± 8.57 (150–191)
Gender, n (%)	
Female	263 (69.8%)
Male	114 (30.2%)
Occupation, n (%)	
Unemployed	23 (6.1%)
Physician	5 (1.3%)
Economist	28 (7.4%)
Laboratory technician	2 (0.5%)
Lecturer/Academic	12 (3.2%)
Secretary	10 (2.7%)
Teacher	8 (2.1%)
Cashier	7 (1.9%)
Accountant	8 (2.1%)
Bank clerk	3 (0.8%)
Nurse	12 (3.2%)
Student	115 (30.5%)
Salesperson	18 (4.8%)
Lawyer	18 (4.8%)
Veterinarian	1 (0.3%)
Dentist	4 (1.1%)
Translator	2 (0.5%)
Retired	7 (1.9%)
Physiotherapist	44 (11.7%)
Driver	2 (0.5%)
Electrician	1 (0.3%)
Mechanic	2 (0.5%)
Hairdresser	6 (1.6%)
Manager	26 (6.9%)
Pharmacist	5 (1.3%)
Cleaner	3 (0.8%)
Waiter	2 (0.5%)
Engineer	3 (0.8%)

**Table 2:** Structural Validity of the ANDI: One-Factor Solution PCA.

**Section A. Sampling Adequacy and Sphericity**		
Kaiser–Meyer–Olkin (KMO)		0.947
Bartlett’s Test of Sphericity χ^2^ (df = 45)		2595.798
*p*-value		<0.001
**Section B. Total Variance Explained (PCA)**	**Component 1**	
Initial Eigenvalue	6.417	
% of Variance (Initial)	64.17%	
Cumulative Variance (Initial)	64.17%	
**Section C. Component Matrix (Unrotated Solution)**	**ANDI Factor 1**	
Work (Puna)	0.876	
Recreation (Rekreacioni)	0.872	
Concentration (Përqendrimi)	0.820	
Pain Intensity (Intensiteti i Dhimbjes)	0.807	
Reading (Leximi)	0.791	
Driving (Drejtimi i makinës)	0.790	
Personal Care (Kujdesi Personal)	0.789	
Headache (Dhimbja e kokës)	0.782	
Sleeping (Fjetja)	0.779	
Lifting (Ngritja e peshave)	0.690	

**Table 3:** Internal Consistency and Test–Retest Reliability, SEM and MDC_95_ per Item of the ANDI (n = 377).

Item	Mean	SD	Item–Total Correlation	ICC (95% CI)	SEM	MDC95
Pain Intensity/ Intensiteti i Dhimbjes	1.12	1.11	0.990	0.990 (0.988–0.992)	0.15	0.41
Personal Care/ Kujdesi Personal	0.72	1.07	0.980	0.990 (0.987–0.992)	0.15	0.41
Lifting/ Ngritja e peshave	1.15	1.19	0.967	0.983 (0.979–0.986)	0.22	0.61
Reading/ Leximi	1.27	1.09	0.983	0.991 (0.989–0.993)	0.14	0.38
Headache/ Dhimbja e kokes	1.53	1.26	0.973	0.986 (0.983–0.986)	0.21	0.57
Concentration/ Përqendrimi	1.28	1.21	0.978	0.989 (0.986–0.991)	0.19	0.53
Work/ Puna	1.03	1.09	0.963	0.981 (0.977–0.985)	0.21	0.58
Driving/ Drejtimi i makinës	1.20	1.26	0.962	0.981 (0.976–0.984)	0.24	0.67
Sleeping/ Fjetja	1.16	1.28	0.984	0.992 (0.990–0.993)	0.13	0.36
Recreation/ Rikrijimi	1.07	1.15	0.975	0.988 (0.985–0.990)	0.18	0.50
Total NDI Score		8.92		0.989	0.94	2.60

Note: All 377 participants completed both test and retest assessments.

## Data Availability

The original contributions presented in this study are included in the article. Further inquiries can be directed to the corresponding author.
